# Synthesis of Siloxyalumoxanes and Alumosiloxanes Based on Organosilicon Diols

**DOI:** 10.3390/molecules22101776

**Published:** 2017-10-20

**Authors:** Galina Shcherbakova, Pavel Storozhenko, Alexander Kisin

**Affiliations:** State Research Institute for Chemistry and Technology of Organoelement Compounds, Shosse Entuziastov 38, Moscow 105118, Russia; info@eos.su (P.S.); kisin@eos.su (A.K.)

**Keywords:** organosiloxyalumoxanes, organoalumosiloxanes, triisobutylaluminum, tetraisobutylalumoxane, diphenylsilanediol, α-diol, γ-diol

## Abstract

We have drawn a few interesting conclusions while studying reaction products of Ph_2_Si(OH)_2_ with Al(*^i^*Bu)_3_ and tetraisobutylalumoxane. In the first place, this is the production (at a Ph_2_Si(OH)_2_ and Al(*^i^*Bu)_3_ equimolar ratio) of an oligomer siloxyalumoxane structure with alternating four- and six-member rings. In addition, it shows isobutyl and phenyl group migration between aluminum and silicon due to the formation of an intramolecular four-member cyclic complex [Ph_2_(OH)SiO]Al(*^i^*Bu)_2_ → [(*^i^*Bu)Ph(OH)SiO]Al(*^i^*Bu)Ph. Ph_2_Si(OH)_2_ interaction with Al(*^i^*Bu)_3_ not only starts from intramolecular complex production, but the chain is terminated for the same reason, which in the case of the Ph_2_Si(OH)_2_ reaction with tetraisobutylalumoxane results in failure of to obtain high-polymer siloxyalumoxane compounds. When Al(*^i^*Bu)_3_ interacts with α- and γ-diols, no oligomer compounds are produced. In the Al(*^i^*Bu)_3_ reaction with α, γ-diols are created in monomer compounds that are likely to have a cyclic structure. Notably, when Al(*^i^*Bu)_3_ interacts with only α-diol, a double excess of Al(*^i^*Bu)_3_ allows for full replacement of hydrogen in the α-diol hydroxyl groups by aluminum alkyl residue with 1,3-bis(diisobutylalumoxymethyl)-1,1,3,3-tetramethyldisiloxane production. At an equimolar ratio of initial reagents, the second isobutyl radical at Al does not interact with the second hydroxyl group of α-diol, apparently due to the steric hindrance, and 1-(diisobutylalumoxymethyl)-3-(hydroxymethyl)-1,1,3,3-tetramethyl-disiloxane is produced. Al(*^i^*Bu)_3_ reactions with γ-diol also result in monomer compounds, but the presence of a chain consisting of three CH_2_-groups between Si and the hydroxyl group facilitates interaction between the second hydroxyl group of γ-diol and the second isobutyl radical Al(*^i^*Bu)_3_. Tetraisobutylalumoxane reactions with α- and γ-diols result in oligomer compounds.

## 1. Introduction

Organosiloxyalumoxane and organoalumoxanesiloxane oligomers were synthesized 30 years ago [1]. Their probable structure from a conventional viewpoint looked like three-coordinated Al atom. These compounds are amorphous, therefore, it did not seem possible to prove their structure by means of X-ray diffraction. However, in the middle of the 1980s, papers dealing with the non-classical structure of alumoxane and alumosiloxane compounds with four-coordinated Al atoms and three-coordinated oxygen atoms were published [2,3,4]. The coordination number of Al atoms in bicyclic and oligomer alumoxanes and alumosiloxanes was shown to be four and may increase to five (or even to six) [2]. The crystalline structure of alumosiloxane of C_8_H_24_Al_3_Br_5_O_6_Si_4_ composition was proved [5]. It was found that the molecule of crystalline alumosiloxane consisted of four condensed nuclei: two planar four-membered rings, built from two aluminum atoms and two oxygen atoms, and two “saddle-shaped” six-membered rings composed of alternating silicon, oxygen, and aluminum atoms. The Al atom, which belongs to all four rings, has a coordination number of five. The remaining atoms of aluminum and silicon have tetrahedral coordination.

Based on the data of [2,3,4,5], the results of [1] were analyzed. In addition, the reaction of Al(*^i^*Bu)_3_ with Ph_2_Si(OH)_2_ in hexadeuterobenzene was studied. The results obtained suggested a probable scheme for the interaction of Al(*^i^*Bu)_3_ with Ph_2_Si(OH)_2_ and the possible structure of the oligomers obtained [6]. However, these results were not published.

Since the beginning of the 1990s, detailed studies of the synthesis, properties, and structure of alkylalumoxanes have been carried out [7,8,9,10,11,12,13,14]. In 2013, works on this problem were summarized in the review [15].

Most closely related to our work was the investigation performed by Andrew R. Barron’s team, they studied the synthesis, properties, and structure of organosiloxyalumoxanes—precursors of alumosilicate ceramics [16,17].

A considerable number of molecular alumosiloxanes and alumosilicates have been obtained using aluminum halogenides, chalcogenides, hydrides, and organometallic compounds as starting materials and reacting them with the appropriate R_n_Si(OH)_4−n_ precursor [18]. The reaction of Al_2_Cl_6_ with an excess of Ph_2_Si(OH)_2_ in THF in the presence of pyridine yielded new anionic and cyclic aluminosiloxanes. The structure of the anionic complex comprises separated pyridinium cations and alumosiloxane anions with a tetrahedral arrangement around the Al atom, which is similar to that in natural aluminosilicates; the core of the cyclic aluminosiloxane is a twelve-membered Al_2_Si_4_O_6_ ring in a chair conformation, which contains a Cl group on each of the two Al atoms [19]. Reaction of Al(*^t^*Bu)_3_ with neol-H_2_ (2,2-dimethylpropane-1,3-diol) yields [Al_2_(*^t^*Bu)_4_(neol-H)_2_]. [Al_2_(*^t^*Bu)_4_(neol-H)_2_] may be considered as a bifunctional (two OH groups), tetradentate (*4O*) ligand as highlighted by its reactivity with Group 13 hydrides and alkyls. Reaction of [Al_2_(*^t^*Bu)_4_(neol-H)_2_] with AlH_3_(NMe_3_), AlH_2_Cl(NMe_3_) and AlMe_3_ yields the tri-aluminum compounds, [Al_3_(*^t^*Bu)_4_(X)(neol)_2_] with X = H, Cl, Me, respectively [20].

The main research on the synthetic and structural chemistry of alumosiloxanes is described in the review [21]. Michael Veith’s works describes the synthesis, physicochemical properties, crystal structure and interaction of polycyclic [Ph_2_SiO]_8_[AlO(OH)]_4_ with various chemical compounds [22,23,24,25].

## 2. Results and Discussion

**Figure 1:**
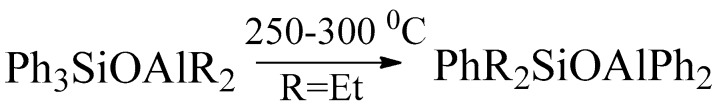
Migration of the phenyl and alkyl groups in triphenylsiloxyaluminumalkyl.

To investigate the mechanism of the Al(*^i^*Bu)_3_ reaction with Ph_2_Si(OH)_2_, we studied (by means of NMR spectroscopy) their interaction in hexadeuterobenzene (C_6_D_6_). In contrast to the reactions in benzene, deuterobenzene solvent makes it possible to determine not only the isobutyl groups, but also benzene’s presence in the ^1^H- and ^13^C-NMR spectra. In the NMR spectra of the reaction mixture after the Al(*^i^*Bu)_3_ interaction with Ph_2_Si(OH)_2_ was complete, a proton signal was observed at 7.23 ppm, and a signal of ^13^C nuclei of unlabeled benzene was observed at 128 ppm [26]. When the siloxyalumoxane oligomer reaction product was released, the produced benzene was distilled along with a solvent (C_6_D_6_) and was recorded in the NMR spectra of the distillate. Benzene may be produced as the result of phenyl and isobutyl groups exchanging between silicon and aluminum. Earlier, a few authors [27] observed phenyl and alkyl group migration in triphenylsiloxyaluminumalkyl at 250–300 °C (Figure 1).

In our case, such migration was observed even at 80 °C.

In ^27^Al-NMR spectra of the products of Al(*^i^*Bu)_3_ reaction with Ph_2_Si(OH)_2_ in C_6_D_6_ at 25 °C broad signal typical for oligomer structures where aluminum coordination number is four was recorded at 61.0 ppm.

**Scheme 1:**
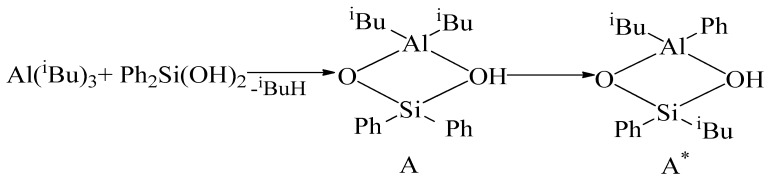
The formation of active intramolecular complex (A*).

According to the abovementioned, we can propose the following mechanism of siloxyalumoxane oligomers production through an active intramolecular complex (A*), where it is probable isobutyl and phenyl groups exchange at aluminum and silicon (Scheme 1).

**Scheme 2:**
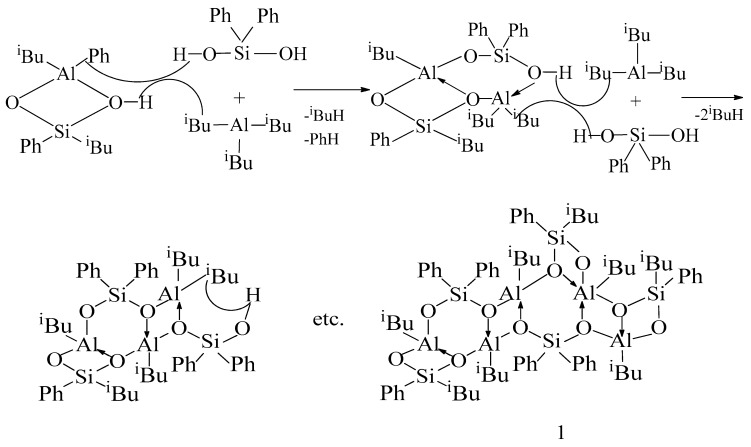
The probable scheme of interaction of Al(*^i^*Bu)_3_ and Ph_2_Si(OH)_2_ at Al:Si = 1:1.

At an equimolar ratio of the starting reagents, hydrogen of the hydroxyl group reacts with the *^i^*Bu- group of another Al(*^i^*Bu)_3_ molecule, and one of the organic groups at aluminum (preferably the phenyl one as it has more electronegative character than the isobutyl group) interacts with hydrogen of OH group of the next Ph_2_Si(OH)_2_ molecule. A six-membered ring is formed; the ring in its turn has active sites able to react with the following Al(*^i^*Bu)_3_ and Ph_2_Si(OH)_2_ molecules, which results in a four-membered alumoxane ring, then a six-membered ring is formed, etc. (Scheme 2).

The chain is terminated due to the intramolecular A* complex.

So, siloxyalumoxane oligomer **1** is formed where one of the phenyl groups is substituted by an isobutyl group at three silicon atoms. Considering the amount of C_6_D_6_ taken for synthesis (at equimolar ratio of Al(*^i^*Bu)_3_:Ph_2_Si(OH)_2_ = 1:1), the distilled C_6_D_6_ must contain about 1.5 wt % of benzene. Mass spectrometric analysis of the distillate showed that along with the used solvent (C_6_D_6_) 1.6 wt % of benzene was distilled.

**Figure 2:**
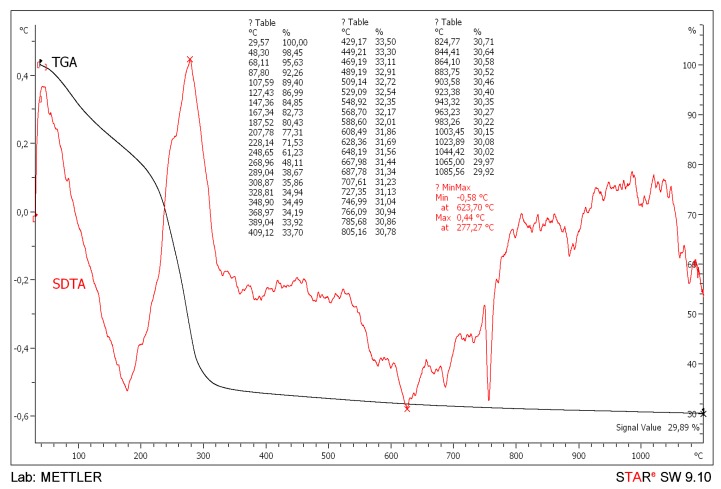
Thermogram of oligomer **1**.

Oligomer **1** production is confirmed by thermo-gravimetric analysis results (Figure 2).

The TGA curve shows that in the temperature range of 30–230 °C, the weight loss is about 30 wt %, while the SDTA curve shows an endo-effect at 180 °C. This corresponds to the complete removal of isobutyl groups in the form of isobutene (theoretically, the weight loss is 31.33 wt %). In the temperature range of 230–650 °C, the weight loss is about 40 wt %, while the SDTA curve shows an exo-effect at 280 °C. This corresponds to the complete removal of the phenyl groups in the form of benzene (theoretically the weight loss is 38.18 wt %). Further pyrolysis in an inert atmosphere (argon) to 1100 °C leads to the formation of an alumosilicate ceramic residue of 29.89 wt %, which actually coincides with the theoretically calculated value for oligomer **1** (30.42 wt %) (Figure 2).

**Scheme 3:**
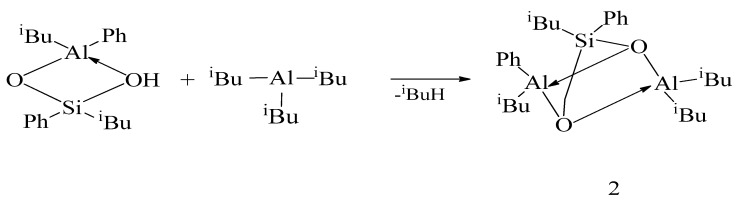
The probable scheme of interaction of Al(*^i^*Bu)_3_ and Ph_2_Si(OH)_2_ at Al:Si = 2:1.

With Al(*^i^*Bu)_3_ (Al:Si = 2:1) excess, the complex is stabilized due to the second Al(*^i^*Bu)_3_ molecule attachment and formation of bis(diisobutylalumoxy)diphenylsilane; taking into account isobutyl and phenyl group migrations between aluminum and silicon, the interaction mechanism can be presented as follows (Scheme 3).

**Scheme 4:**
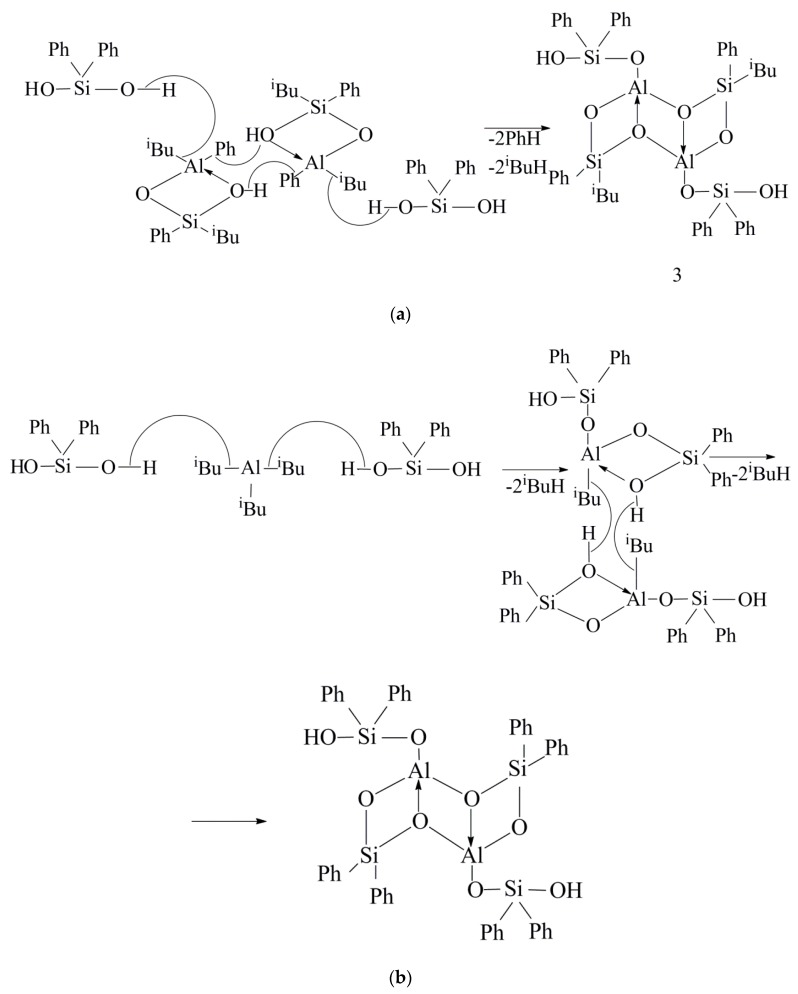
The probable scheme of interaction of Al(*^i^*Bu)3 and Ph_2_Si(OH)_2_ at Al:Si = 1:2.

When the reaction proceeds at Ph_2_Si(OH)_2_ (Al:Si = 1:2) excess, organosiloxyalumoxane formation may be presented in two ways (Scheme 4a,b).

Scheme 4a is the most probable. This is confirmed by the fact that in the ^1^H-NMR spectra of siloxyalumoxane with molar ratio Al:Si = 1:2, low resolution signals of isobutyl group protons at 1.05 ppm of CH_3_(*^i^*Bu–Si) and 1.4 ppm of CH_2_(*^i^*Bu–Si) were observed. Moreover, if one mole of Al(*^i^*Bu)_3_ interacts with two moles of Ph_2_Si(OH)_2_, the amount of gas released is much less as compared to the theory and amounts to less than 70%, which corresponds to two isobutyl groups migrating from aluminum to silicon.

**Figure 3:**
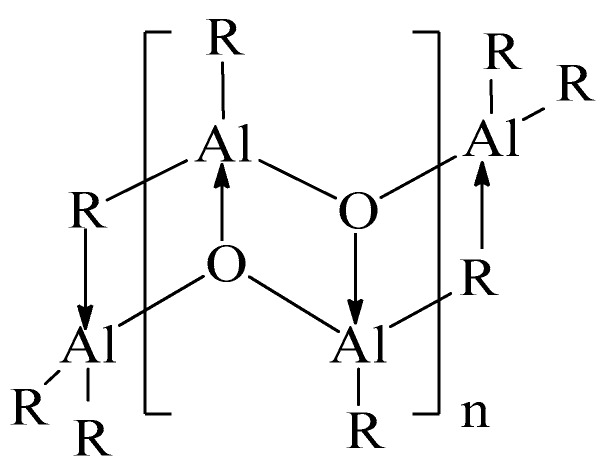
Schematic structural formula of alkylalumoxanes.

Considering alkylalumoxane reactions with Ph_2_Si(OH)_2_, we kept in mind that the aluminum atom is four-coordinated in alkylalumoxanes [2,3,4,5,6,7,8,9,10,11,12,13,14,15], whose schematic structural formulas are presented as follows: (at *n* = 1—tetraisobutylalumoxane, at *n* > 1—polyisobutylalumoxane) [3] (Figure 3).

**Scheme 5:**
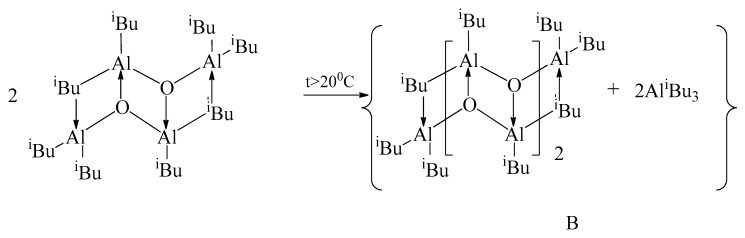
Disproportionation of tetraisobutylalumoxane.

It should be noted that at a temperatures above 20 °C tetraisobutylalumoxane disproportionation takes place with triisobutylaluminum release [3,13] (Scheme 5).

**Scheme 6:**
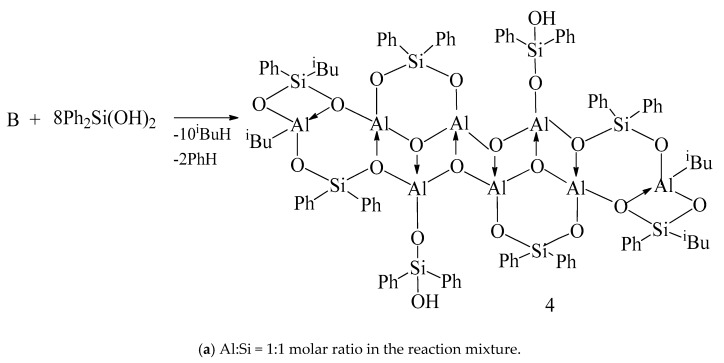

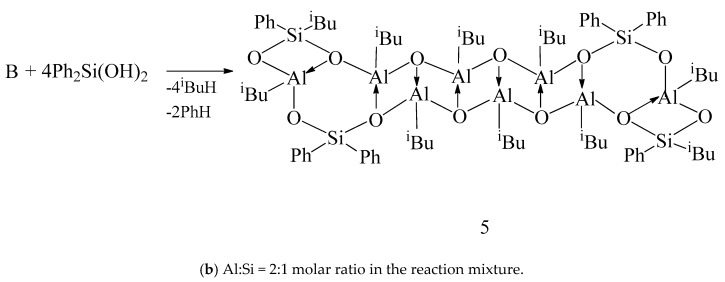
Probable scheme of interaction of tetraisobutylalumoxane with Ph_2_Si(OH)_2_.

At a temperature of 80 °C, a mixture of triisobutylaluminum and pentaisobutyltrialumoxane (B) reacts with Ph_2_Si(OH)_2_, therefore, a part of Ph_2_Si(OH)_2_ interacts with Al(*^i^*Bu)_3_ forming the intramolecular A* complex. The other part of Ph_2_Si(OH)_2_ reacts with the double alumoxane chain, the alumoxane chain, therewith, is terminated by the A* complex, which means it is impossibile to obtain high-polymer siloxyalumoxane compounds from tetraisobutylalumoxane and Ph_2_Si(OH)_2_ (Scheme 6).

Siloxyalumoxane oligomers **4** or **5** containing four- and six-membered rings were produced.

Siloxyalumoxanes **1**–**5** were white, glassy, benzene-soluble oligomers with different molecular weights.

**Figure 4:**
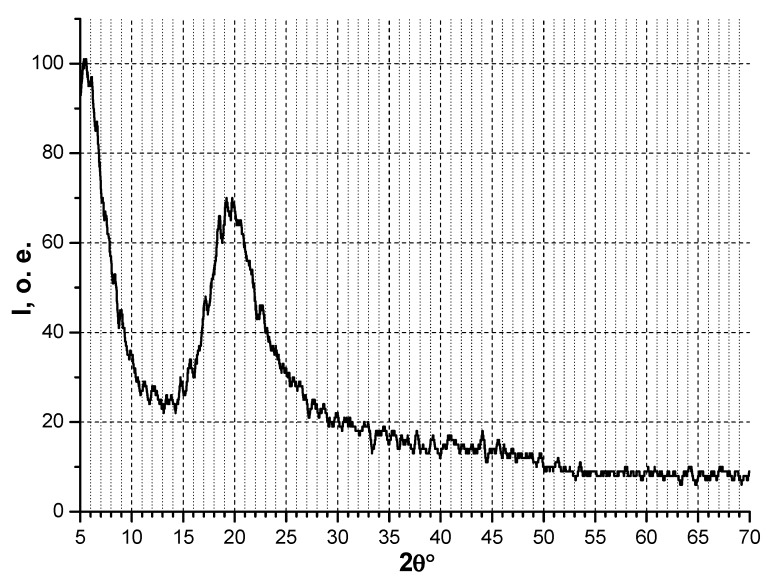
Typical diffractogram of siloxyalumoxane oligomers (**1**–**6**).

X-ray amorphism of siloxyalumoxane oligomers (Figure 4) did not allow for the determination of their crystalline characteristics, but the presence of reduced structural units in oligomers (**1**)–(**5**) was confirmed by multinuclear magnetic resonance, IR spectroscopy, TGA, and elemental analysis.

In contrast to the starting Al(*^i^*Bu)_3_, tetraisobutylalumoxane, and Ph_2_Si(OH)_2_, their interaction products in the IR spectra have highly intense broad absorption bands in the region of 1050–1075 cm^−1^, typical for Al–O–Si bonds, as well as an absorption band of medium intensity in the region of 820–840 cm^−1^, corresponding to valence vibrations of Al–O–Al bond (bridge). Presence or absence of a wide absorption band in the region of 3400–3500 cm^−1^, typical for hydroxyl group valence vibrations, confirms probable oligomer **1**–**5** structures.

In ^1^H-NMR spectra of the reaction mixture after the completion of the Al(*^i^*Bu)_3_ (tetraisobutylalumoxane) reaction with Ph_2_Si(OH)_2_ in benzene, isobutyl group proton signals were observed as low resolution multiplets in the region of 0.5 to 2.5 ppm.

^1^H-NMR spectrum of the concentrated isobutylalumoxanephenylsiloxane oligomer in C_6_D_6_ had a broadened signal at 7.5 ppm, typical for phenyl group protons, and another signal at 1.5 ppm, corresponding to isobutyl group protons.

Therefore, an organoaluminum compound reaction with diphenylsilanediol results in organosiloxyalumoxane compounds with alternating four- and six-membered rings containing aluminum atoms with the coordination number equal to four. Isobutyl and phenyl groups at aluminum and silicon may migrate in the process of reaction.

The structure of the alumoxanesiloxane oligomers is confirmed by [16,17] where it was shown that the hydrolytically stable alumoxanes of the formula [Al(O)_x_(OH)_y_(X)_z_]_n_ (X = OSiR_3_, O_2_CR) are neither linear nor ring structures, but are spatial clusters. These alumoxanes have an isostructural center (nucleus) similar to minerals such as boehmite and diaspore {[Al(O)(OH)]_n_}, wherein central aluminum atoms are six-coordinated. In the case of siloxy derivatives, four-coordinated aluminum atoms are present circumferentially [16,17].

Triisobutylaluminum and tetraisobutylalumoxane reactions with α-diol and γ-diol were conducted in benzene at different temperatures (20 °C and 80 °C) and given molar ratios Al:Si (1:1; 2:1; 3:1; 1:2). Al(*^i^*Bu)_3_ interaction with α- and γ-diols occurred at 20 °C, but full gas release was observed only at 80 °C. The resulting products were viscous colorless or yellow clear liquids, readily soluble in hydrocarbon solvents and readily hydrolysable in air [1,6].

Elemental analysis data as well as molecular weight of the synthesized compounds suggest that depending on mole ratio of the starting reagents, Al(*^i^*Bu)_3_ reactions with α, γ-diols result in compounds (**6**–**11**), probably having cyclic structure. Moreover, when Al(*^i^*Bu)_3_ interacts with α-diol, only a double excess of Al(*^i^*Bu)_3_ allows for full replacement of the hydroxyl group hydrogen in α-diol with aluminumalkyl residue to form 1,3-bis(diisobutylalumoxymethyl)-1,1,3,3-tetramethyldisiloxane (**7**). At an equimolar ratio of the starting reagents, the second isobutyl radical at Al does not react with the second hydroxyl group of α-diol, probably due to steric hindrance, and 1-(diisobutylalumoxymethyl)-3-(hydroxymethyl)-1,1,3,3-tetramethyldisiloxane (**6**) is produced.

**Scheme 7:**
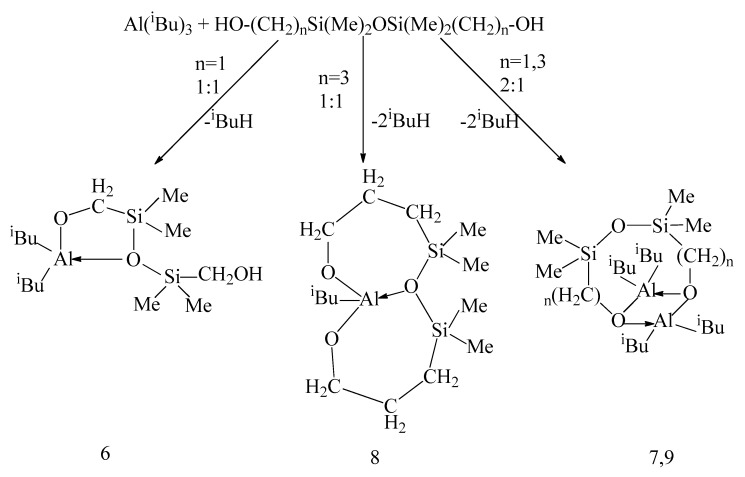
Probable scheme of interaction of Al(*^i^*Bu)_3_ with α, γ-diols.

Al(*^i^*Bu)_3_ reactions with γ-diol also result in monomer compounds (**8**,**9**), but the presence of a chain comprising three CH_2_-groups between the *Si* atom and a hydroxyl group simplifies the interaction between the second hydroxyl group of γ-diol and the second isobutyl radical Al(*^i^*Bu)_3_. Probable triisobutylaluminum reactions with α, γ-diols are presented by Scheme 7.

The comparison of the IR spectra of 1-(diisobutylalumoxymethyl)-3-(hydroxymethyl)-1,1,3,3-tetramethyldisiloxane (**6**) and 1,3-bis(diisobutylalumoxymethyl)-1,1,3,3-tetramethyldisiloxane (**7**) with the IR spectrum of the starting α-diol confirms our assumptions. In the IR spectrum of compound (**6**), there is a wide absorption band in the region of 660 cm^−1^ corresponding to valence vibrations ν(Al–C), and the intensity of an absorption band in the region of 3400 cm^−1^ ν(OH) significantly decreases. In the IR spectrum of compound (**7**), there is no absorption in the region of 3400 cm^−1^, and an intense absorption band in the region of 665 cm^−1^ ν(Al–C) appears. Moreover, in both cases, the intense absorption band broadens in the region of 1060 cm^−1^ ν(Si–O–Si) and has a shoulder of 1015 cm^−1^ ν(Al–O–C).

In contrast to the ^1^H-NMR spectrum of the starting α-diol, wherein proton signals at 0.38 ppm (CH_3_), 3.5 ppm (CH_2_), and 4.62 ppm (OH) were recorded in the spectra of compounds (**6**,**7**) and proton signals of isobutyl groups were observed at 0.4 ppm (CH_2_), 1.07 ppm (CH_3_), and 1.45 ppm (CH), but the proton signal of the CH_2_ group of α-diol was shifted to a weaker field and was manifested at 3.65 ppm.

Comparison of the IR spectra of 2-isobutyl-7,7,9,9-tetramethyl-1,3,8-trioxa-7,9-disila-2-alumacyclododecane (**8**) and 1,3-bis(diisobutylalumoxypropyl)-1,1,3,3-tetramethyldisiloxane (**9**) with the IR spectrum of the starting γ-diol shows that there is no absorption band in the region of 3400 cm^−1^ typical for ν(OH), but a broad intense band appears in the region of 670 cm^−1^, typical for valence vibrations of ν(Al–C) bonds, as well, a shoulder in the region of 1010–1020 cm^−1^, corresponding to ν(Al–O–C) vibrations, is clearly manifested.

The ^1^H-NMR spectrum of the γ-diol has proton signals at 0.25 ppm (CH_3_), 0.75 ppm (α-CH_2_), 1.8 ppm (β-CH_2_), 3.75 ppm (γ-CH_2_), and 4.4 ppm (OH). In the ^1^H-NMR spectra of compounds **8** and **9**, signals corresponding to isobutyl group protons and γ-diol protons are overlapped.

Tetraisobutylalumoxane (B) reactions with α- and γ-diols result in oligomer compounds, but here also, as in the case with diphenylsilanediol, molecular weight growth is due to isobutyl radicals replacement with oxyorganosiloxane groupings. The resulting oligomers are white, glassy products, readily soluble in benzene. Compounds synthesized from tetraisobutylalumoxane and α-diol dissolved in heptane as well, but compounds synthesized from tetraisobutylalumoxane and γ-diol did not dissolve in heptane.

**Scheme 8:**
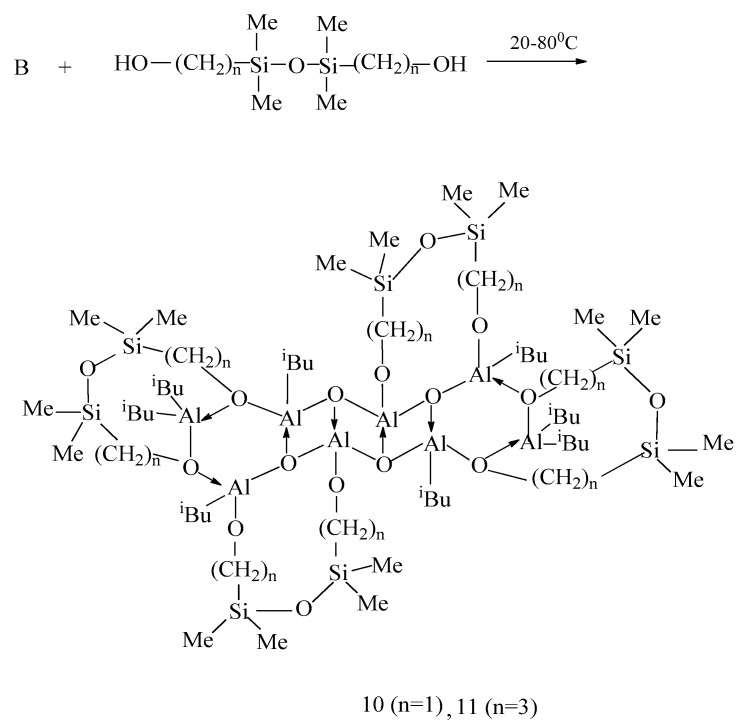
Probable scheme of interaction of isobutylalumoxane with α, γ-diols.

The formation of alumoxanes comprising oxyorganosiloxane fragments may be presented by Scheme 8.

The ^1^H-NMR and IR spectra of the reaction products of tetraisobutylalumoxane reacted with α- and γ-diols were similar to those of the compounds produced by Al(*^i^*Bu)_3_ reactions with α- and γ-diols. They confirmed hydroxyl group absence in the resulting compounds and the presence of isobutyl groups, Al–C, Al–O–C bonds, and oxyorganosiloxane fragments. The molecular weight of the synthesized oxyorganosiloxane-containing alumoxanes depended on the molecular weight of the starting alkylalumoxane.

## 3. Experimental

### 3.1. General Methods

Triisobutylaluminum, tetraisobutylalumoxane, diphenylsilanediol, 1,3-bis(hydroxymethyl)-1,1,3,3-tetramethyldisiloxane (α-diol), and 1,3-bis(hydroxypropyl)-1,1,3,3-tetramethyldisiloxane (γ-diol) were purchased at Pilot Plant in Redkino. The solvent (benzene-*d*_6_) used was acquired from “Galakhim” JSC (Moscow, Russia). The solvent (benzene) used was purchased at “Component-Reaktiv” JSC. All synthesis processes were performed in dry argon (the content of O_2_ and H_2_O <0.005 wt %). The aluminum content was determined by trilonometry. Silicon content was determined spectrophotometrically as a silicon molybdenum complex at a wavelength of 400 nm. Carbon and hydrogen were determined gravimetrically by burning a weighed sample in oxygen flow. The content of hydroxyl groups in the organosiloxyalumoxanes and organoalumosiloxanes was found by a gasometric method. Molecular weights of the obtained compounds were determined by the cryoscopic method. Gas volume released in the reactions was determined by gas volumetric method. Mass spectrometric analysis of the distillate was conducted on Agilent 240 GC mass-spectrometer/MS Ion Trap at ionizing voltage of 70 eV. The IR spectra of organosiloxyalumoxanes and organoalumosiloxanes were recorded on a Perkin-Elmer spectrometer, model 180 within the range of 400–4000 cm^−1^. The ^1^H- and ^27^Al-NMR spectra of organosiloxyalumoxanes were obtained using a Bruker AM-360 NMR spectrometer (360 MHz for ^1^H-NMR and 93.8 MHz for ^27^Al-NMR) using solutions in C_6_D_6_. [Al(H_2_O)_6_]^3+^ was used as an external standard. ^1^H-NMR spectra of organosilicondiols 1,3-bis-(hydroxymethyl)-1,1,3,3-tetramethyldisiloxane (α-diol) and 1,3-bis-(hydroxypropyl)-1,1,3,3-tetramethyldisiloxane (γ-diol) were obtained using a Varian T-60A NMR spectrometer (60 MHz for ^1^H-NMR) using solutions in C_6_D_6_. Tetramethylsilane was used as an internal standard. Phase compositions were determined by X-ray diffraction at room temperature on a Shimadzu XRD-6000 vertical X-ray diffractometer with CuKα radiation (λ_Kαcp_ = (2λ_Kα1_ + λ_Kα2_)/3 = 1.54178 [Å]). The crystalline and amorphous phases present were identified using ICDD PDF (The International Centre for Diffraction Data Powder Diffraction File) Release 2003 data.

### 3.2. Synthetic Procedures

#### 3.2.1. General Procedure for the Preparation of Organosiloxyalumoxanes

The interaction of organoaluminum compounds (OAC)-Al(*^i^*Bu)_3_ and tetraisobutylalumoxane with diphenylsilanediol Ph_2_Si(OH)_2_ was carried out in deuterobenzene C_6_D_6_. Ph_2_Si(OH)_2_ suspension in C_6_D_6_ was heated to 60–80 °C. The calculated amount of OAC (10–20 wt %, solution in deuterobenzene) was added under stirring, and isobutane (*^i^*BuH) gas was released therewith; the gas was collected in a gas meter. Upon completion of OAC feed the reaction mixture was held for 1–2 h at 80 °C, and then it was cooled to room temperature. The reaction mass was a colorless, transparent liquid. The solvent was distilled thereafter. Light yellow or white solid glassy products were obtained. The yield of the target products was 85–99 wt %. Specific reaction conditions are presented in Table 1.

#### 3.2.2. General Procedure for the Preparation of Organoalumosiloxanes and Organoalumoxanesiloxanes

The interaction of organoaluminum compounds (OAC) − Al(*^i^*Bu)_3_ and tetraisobutylalumoxane with 1,3-bis(hydroxymethyl)-1,1,3,3-tetramethyldisiloxane (α-diol) and with 1,3-bis(3-hydroxypropyl)-1,1,3,3-tetramethyldisiloxane (γ-diol) was performed in benzene. A 20 wt % solution of α-diol (γ-diol) in benzene was prepared. Under stirring at a temperature of 20 °C, a calculated amount of OAC (20 wt %, solution in benzene) was added, depending on the specified Al:Si (1:1; 2:1; 1:2) mole ratio. Then, the reaction mass was heated to 80 °C and held at 80 °C under stirring (after gas release completion) for an hour. The yield of desired products amounted to 74–100 wt %. Specific reaction conditions are presented in Table 2.

*[(^i^BuPhSiO)_3_(PhSiO)_2_(^i^BuAlO)_5_]* (**1**). Yield: 94.0%; ^1^H-NMR (360 MHz, C_6_D_6_): broad m 0.5–2.5 ppm (*^i^*Bu); broad m 6.7–8.0 ppm (aromatic protons); ^27^Al-NMR (93.8 MHz, C_6_D_6_): broad 61 ppm; IR (cm^−1^), 2970–2850 ν(C–H); 1470, 1380 δ(C–H); 1130 ν(ring C_6_H_5_); 1070 ν(Si–O–Al); 820 ν(Al–O–Al); 720, 700 (Si–Ph); 670 ν(Al–C); 520, 480; Anal. Calculated for C_74_H_107_Al_5_Si_5_O_10_: M_n_, 1430; C, 62.10; H, 7.48; Al, 9.44; Si, 9.79, Found: M_n_, 1470; C, 60.15; H, 7.47; Al, 9.24; Si, 9.89.

*[^i^BuPhSiO_2_Al_2_(^i^Bu)_3_Ph]* (**2**). Yield: 99.3%; ^1^H-NMR (60 MHz, C_6_D_6_): broad m 0.7 ppm CH_2_(*^i^*Bu–Al); broad m 1.2 ppm CH_3_(*^i^*Bu–Al); broad m 1.6 ppm CH_3_(*^i^*Bu–Si); broad m 1.8 ppm CH(*^i^*Bu–Si); IR (cm^−1^), 2960–2840 ν(C–H); 1470, 1435, 1380 δ(C–H), 1120 ν(ring C_6_H_5_), 1070 ν(Si–O–Al); 830 ν (Al–O–Al); 740, 720, 700 (Si–Ph); 670 ν(Al–C); 520, 480; Anal. Calculated for C_28_H_46_Al_2_SiO_2_: M_n_, 496; C, 67.74; H, 9.27; Al, 10.89; Si, 5.65, Found: M_n_, 504; C, 67.21; H, 9.08; Al, 10.80; Si, 5.60.

*[(^i^BuPhSiO)_2_(Ph_2_Si(OH)O)_2_(Al_2_O_2_)]* (**3**). Yield: 86.3%; ^1^H-NMR (60 MHz, C_6_D_6_): 1.05 ppm, d, *J* = 6 Hz, CH_3_(*^i^*Bu–Si); 1.4 ppm d, *J* = 6 Hz, CH_2_(*^i^*Bu–Si); IR (cm^−1^), 3300 ν(O–H); 2980–2860 ν(C–H); 1470, 1380 δ(C–H); 1120 ν(ring C_6_H_5_); 1070 ν(Si–O–Al); 1030 ν(Si–O) 820 ν(Al–O–Al); 720, 700 (Si–Ph); 520, 480; Anal. Calculated for C_44_H_54_Al_2_Si_4_O_8_: M_n_, 872; C, 60.55; H, 6.19; Al, 6.19; Si, 12.84; OH, 3.90, Found: M_n_, 920; C, 60.22; H, 6.36; Al, 6.50; Si, 12.90; OH, 4.20.

*[(^i^BuPhSiO)_2_(Ph_2_SiO_2_)_4_(Ph_2_Si(OH)O)_2_(^i^BuAl_2_O)_2_(Al_2_O_2_)_2_]* (**4**). Yield: 99.6%; ^1^H-NMR (60 MHz, C_6_D_6_): 1.05 ppm, d, *J* = 6 Hz, CH_3_ (*^i^*Bu–Al); 1.8 ppm m, CH_3_(*^i^*Bu–Si); IR (cm^−1^), 3350 ν(O–H); 2970–2860 ν(C–H); 1460, 1375 δ(C–H); 1120 ν(ring C_6_H_5_); 1070–1020 ν(Si–O–Al); 810 ν(Al–O–Al); 710, 690 (Si–Ph); 670 ν(Al–C); 520, 480; Anal. Calculated for C_100_H_108_Al_8_Si_8_O_20_: M_n_, 2068; C, 58.03; H, 5.22; Al, 10.44; Si, 10.83; OH, 1.40, Found: M_n_, 2100; C, 57.83; H, 5.17; Al, 10.10; Si, 10.47, OH, 1.64.

*[(^i^BuPhSiO)_2_(Ph_2_SiO_2_)_2_(^i^Bu_2_Al_2_O)_2_(^i^Bu_2_Al_2_O_2_)_2_]* (**5**). Yield: 99.5%; ^1^H-NMR (60 MHz, C_6_D_6_): 1.1 ppm, d, *J* = 6 Hz, CH_3_(*^i^*Bu–Al); 1.35 ppm d, *J* = 7 Hz, CH_3_(*^i^*Bu–Si); 2.3 ppm, m, CH_3_(*^i^*Bu–Al); IR (cm^−1^), 2980–2860 ν(C–H); 1470, 1380 δ(C–H); 1130 ν(ring C_6_H_5_); 1070–1030 ν(Si–O–Al); 805 ν(Al–O–Al); 720, 700 (Si–Ph); 670 ν(Al–C); 520, 480; Anal. Calculated for C_76_H_120_Al_8_Si_4_O_12_: M_n_, 1552; C, 58.76; H, 7.73; Al, 13.92; Si, 7.22, Found: M_n_, 1520; C, 58.13; H, 7.96; Al, 14.19; Si, 7.62.

*1-(Diisobutylalumoxymethyl)-3-(hydroxymethyl)-1,1,3,3-tetramethyldisiloxane* (**6**). Yield: 74.0%; IR (cm^−1^), 3400 ν(O–H); 2980, 2860 ν(C–H); 1470, 1395, 1370 δ(C–H); 1260 ν(Si–CH_3_); 1060–1015 ν(Si–O–Si); ν(C–O–Al); 860, 800 ν(O–Si–(CH_3_)_2_); 665 ν(Al–C); Anal. Calculated for C_14_H_35_AlSi_2_O_3_: M_n_, 334; C, 50.30; H, 10.48; Al, 8.08; Si, 16.77; OH, 5.09, Found: M_n_, 330; C, 50.63; H, 10.59; Al, 8.30; Si, 16.30, OH, 5.20.

*1,3-bis(Diisobutylalumoxymethyl)-1,1,3,3-tetramethyldisiloxane* (**7**). Yield: 95.5%; ^1^H-NMR (60 MHz, C_6_D_6_): 0.4 ppm, s, ((CH_3_)_2_Si, α-diol); 0.5 ppm, broad m, (CH_2_,*^i^*Bu, olyg); 1.05 ppm, d, *J* = 6 Hz, CH_3_(*^i^*Bu–Al); 1.5 ppm, broad m, CH(*^i^*Bu–Al, olyg); 3.7 ppm, s, (CH_2_O-α-diol); IR (cm^−1^), 2980, 2860 ν(C–H); 1465, 1395, 1360 δ(C–H); 1260 ν(Si–CH_3_); 1060–1015 ν(Si–O–Si); ν(C–O–Al); 860, 800 (O–Si–(CH_3_)_2_); 665 ν(Al–C); Anal. Calculated for C_22_H_52_Al_2_Si_2_O_3_: M_n_, 474; C, 55.70; H, 10.97; Al, 11.39; Si, 11.81, Found: M_n_, 480; C, 55.42; H, 10.34; Al, 11.10; Si, 11.40.

*2-Isobutyl-7,7,9,9-tetramethyl-1,3,8-trioxa-7,9-disila-2-alumacyclododecane* (**8**). Yield: 85.0%; IR (cm^−1^), 2980, 2860 ν(C–H); 1465, 1380, 1360, 1320 δ(C–H); 1255 ν(Si–CH_3_); 1060–1010 ν(Si–O–Si); ν(C–O–Al); 860, 800 ν(O–Si–(CH_3_)_2_); 670 ν(Al–C); Anal. Calculated for C_14_H_33_AlSi_2_O_3_: M_n_, 332; C, 50.60; H, 9.94; Al, 8.13; Si, 16.87, Found: M_n_, 330; C, 48.98; H, 9.90; Al, 7.94; Si, 16.46.

*1,3-bis(Diisobutylalumoxypropyl)-1,1,3,3-tetramethyldisiloxane* (**9**). Yield: 93.4%; ^1^H-NMR (60 MHz, C_6_D_6_): 0.3 ppm, s, ((CH_3_)_2_Si, γ-diol); 0.5 ppm, broad m, (CH_2_,*^i^*Bu, olyg); 1.05 ppm, d, *J* = 6 Hz, CH_3_(*^i^*Bu–Al); 1.4 ppm, broad m, (α-CH_2_, γ-diol); 2 ppm, broad m, (β-CH_2_, γ-diol); 3.7 ppm, broad m, (γ-CH_2_, γ-diol); IR (cm^−1^), 2980, 2860 ν(C–H); 1465, 1400, 1365, 1320 δ(C–H); 1255 ν(Si–CH_3_); 1060–1020 ν(Si–O–Si); ν(C–O–Al); 840, 800 ν(O–Si–(CH_3_)_2_); 670 ν(Al–C); Anal. Calculated for C_26_H_60_Al_2_Si_2_O_3_: M_n_, 530; C, 58.87; H, 11.32; Al, 10.19; Si, 10.57, Found: M_n_, 500; C, 58.54; H, 11.00; Al, 9.90; Si, 10.10.

*{[Me_4_(O)Si_2_(CH_2_)_2_O_2_]_4_(^i^Bu_2_Al)_2_(^i^BuAl)_2_(^i^BuAl_2_O_2_)_2_}* (**10**). Yield: 99.9%; IR (cm^−1^), 2980, 2860 ν(C–H); 1460, 1395, 1360 δ(C–H); 1255 ν(Si–CH_3_); 1060–1020 ν(Si–O–Si); ν(C–O–Al); 860, 800 (O–Si–(CH_3_)_2_); 750 ν(Al–O–Al); 660 ν(Al–C); Anal. Calculated for C_56_H_136_Al_8_Si_8_O_16_: M_n_, 1504; C, 44.68; H, 9.04; Al, 14.36; Si, 14.89, Found: M_n_, 1490; C, 44.34; H, 9.23; Al, 14.70; Si, 15.10.

*{[Me_4_(O)Si_2_(CH_2_CH_2_CH_2_)_2_O_2_]_4_(^i^Bu_2_Al)_2_(^i^BuAl)_2_(^i^BuAl_2_O_2_)_2_}* (**11**). Yield: 99.3%; IR (cm^−1^), 2980, 2860 ν(C–H); 1465, 1380, 1360, 1320 δ(C–H); 1260 ν(Si–CH_3_); 1065–1015 ν(Si–O–Si); ν(C–O–Al); 840, 800, 770 (O–Si–(CH_3_)_2_), ν(Al–O–Al); 670 ν(Al–C); Anal. Calculated for C_72_H_168_Al_8_Si_8_O_16_: M_n_, 1728; C, 50.00; H, 9.72; Al, 12.50; Si, 12.96, Found: M_n_, 1680; C, 49.19; H, 9.31; Al, 12.10; Si, 12.60.

## 4. Conclusions

Triisobutylaluminum interaction with diphenylsilanediol results in oligomer products only at an equimolar ratio of the starting Al(*^i^*Bu)_3_ and Ph_2_Si(OH)_2_. During the reaction, the isobutyl and phenyl groups at aluminum and silicon may migrate. In the interaction of alkylalumoxanes with diphenylsilanediol, the increase in molecular weight occurs not due to chain growth, but only due to the replacement of alkyl radicals by diphenylsilanol groups. When Al(*^i^*Bu)_3_ interacts with α- and γ-diols, no oligomer compounds are produced. Tetraisobutylalumoxane reaction with α- and γ-diols results in oligomer compounds, and yet, as in the case of diphenylsilanediol, the increase in the molecular weight is due only to the replacement of isobutyl radicals by oxyorganosiloxane groupings.

## Figures and Tables

**Table 1:** Organoaluminum compounds (OAC) interaction with Ph_2_Si(OH)_2_. (Reaction conditions: solvent—hexadeuterobenzene, T = 80 °C, τ = 1–2 h).

No.	OAC	Al:Si Molar Ratio in the Reaction Mixture	Amount of Gas Released in the Reaction, mL		Product Yield, wt %
			Calculated	Experiment	
1	Al(*^i^*Bu)_3_	1:1	223.28	210	94.0
2	Al(*^i^*Bu)_3_	2:1	77.75	80	99.3
3	Al(*^i^*Bu)_3_	1:2	299.04	258	86.3
4	tetraisobutylalumoxane	1:1	279.11	280	99.6
5	tetraisobutylalumoxane	2:1	93.70	93	99.3

**Table 2:** OAC interaction with α- and γ-diols. (Reaction conditions: solvent—benzene, T = 20–80 °C, τ = 1–2 h).

No.	OAC	Organosilicon compounds (OSC)	Molar Ratio		Amount of Gas Released in the Reaction, mL		Product Yield, wt %
			OAC:OSC	Al:Si	Calculated	Experiment	
6	Al(*^i^*Bu)_3_	α-diol	1:1	1:2	155.50	115	74.0
7	Al(*^i^*Bu)_3_	α-diol	2:1	1:1	75.75	76	95.5
8	Al(*^i^*Bu)_3_	γ-diol	1:1	1:2	239.23	203	85.0
9	Al(*^i^*Bu)_3_	γ-diol	2:1	1:1	207.30	194	93.4
10	tetraisobutylalumoxane	α-diol	1:1	1:1	149.52	150	99.9
11	tetraisobutylalumoxane	γ-diol	1:1	1:1	193.70	200	99.3
